# Subacute thyroiditis following COVID-19: A systematic review

**DOI:** 10.3389/fendo.2023.1126637

**Published:** 2023-04-05

**Authors:** Elahe Meftah, Rahem Rahmati, Fatemeh Zari Meidani, Sanaz Khodadadi, Kosar Chitzan-Zadeh, Fatemeh Esfahanian, Shiva Afshar

**Affiliations:** ^1^ Students’ Scientific Research Center, Tehran University of Medical Sciences, Tehran, Iran; ^2^ Students Research Committee, Shahrekord University of Medical Sciences, Shahrekord, Iran; ^3^ Students Research Committee, Tehran Medical Sciences Branch, Islamic Azad University, Tehran, Iran; ^4^ Students Research Committee, Ahvaz Jundishapur University of Medical Sciences, Ahvaz, Iran; ^5^ Department of Endocrinology, Vali-Asr Hospital, Imam Khomeini Hospital Complex, Tehran University of Medical Sciences, Tehran, Iran; ^6^ School of Medicine, Dezful University of Medical Sciences, Dezful, Iran

**Keywords:** subacute thyroiditis (SAT), De Quervain thyroiditis, COVID-19, SARS-CoV-2, thyroid, long-term COVID-19 symptoms, long COVID, systematic review

## Abstract

**Background:**

Subacute thyroiditis (SAT) is a self-limiting thyroid inflammatory disease occurring specifically after upper respiratory tract infections. Since COVID-19 is a respiratory disease leading to multi-organ involvements, we aimed to systematically review the literature regarding SAT secondary to COVID-19.

**Methods:**

We searched Scopus, PubMed/MEDLINE, Cochrane, Web of Science, ProQuest, and LitCovid databases using the terms “subacute thyroiditis” and “COVID-19” and their synonyms from inception to November 3, 2022. We included the original articles of the patients with SAT secondary to COVID-19. Studies reporting SAT secondary to COVID-19 vaccination or SAT symptoms’ manifestation before the COVID-19 infection were not included.

**Results:**

Totally, 820 articles were retained. Having removed the duplicates, 250 articles remained, out of which 43 articles (40 case reports and three case series) with a total of 100 patients, were eventually selected. The patients aged 18–85 years (Mean: 42.70, SD: 11.85) and 68 (68%) were women. The time from the onset of COVID-19 to the onset of SAT symptoms varied from zero to 168 days (Mean: 28.31, SD: 36.92). The most common symptoms of SAT were neck pain in 69 patients (69%), fever in 54 (54%), fatigue and weakness in 34 (34%), and persistent palpitations in 31 (31%). The most common ultrasonographic findings were hypoechoic regions in 73 (79%), enlarged thyroid in 46 (50%), and changes in thyroid vascularity in 14 (15%). Thirty-one patients (31%) were hospitalized, and 68 (68%) were treated as outpatients. Corticosteroids were the preferred treatment in both the inpatient and outpatient settings (25 inpatients (81%) and 44 outpatients (65%)). Other preferred treatments were nonsteroidal anti-inflammatory drugs (nine inpatients (29%) and 17 outpatients (25%)) and beta-blockers (four inpatients (13%) and seven outpatients (10%)). After a mean duration of 61.59 days (SD: 67.07), 21 patients (23%) developed hypothyroidism and thus, levothyroxine-based treatment was used in six of these patients and the rest of these patients did not receive levothyroxine.

**Conclusion:**

SAT secondary to COVID-19 seems to manifest almost similarly to the conventional SAT. However, except for the case reports and case series, lack of studies has limited the quality of the data at hand.

## 1 Introduction

Although COVID-19 was initially considered a respiratory tract disease, it is currently identified as a systemic infection affecting multiple organs. Some organs that may be affected by COVID-19 are the respiratory, cardiovascular, hematopoietic, gastrointestinal, urinary, nervous, musculoskeletal, and endocrine systems (1). Despite this fact that several preventive measures have brought the COVID-19 pandemic almost under control, there are some uncertainties regarding the potentially prolonged sequela of COVID-19.

A significant proportion of COVID-19 patients report symptoms known as long-term COVID-19 symptoms that persist long after recovery. The term “long-term COVID-19” or “long COVID” refers to lack of complete recovery within two or three weeks after the initial manifestations of COVID-19 (2). Multi-organ involvement is responsible for long-term COVID-19 symptoms, including fatigue, dyspnea, alopecia, hyperhidrosis, insomnia, anxiety, arthralgia, weight loss, fever, body pains, carditis, and persistent reduction of lung function (3), some of which are common among other conditions, such as subacute thyroiditis. This overlap might result in misdiagnosing those other conditions as long-term COVID-19 symptoms and the patients receiving ill-suited managements (4).

Subacute thyroiditis (SAT) is a self-limiting thyroid inflammatory disease characterized by symptoms like anterior neck pain that may radiate to the ears and jaw (4). The potential systemic and laboratory findings of SAT are low-grade fever, fatigue, elevated C-reactive protein (CRP), increased erythrocyte sedimentation rate (ESR), and the suppression of thyroid-stimulating hormone (TSH) (5). Thyroid gland tenderness and small diffuse goiter may be detected in the physical examination. The clinical course of SAT is divided into three phases: thyrotoxicosis, hypothyroidism, and normal thyroid function. At the time of diagnosis, most patients display mild to moderate thyrotoxicosis manifestations, such as weight loss, palpitations, and tremors (6).

SAT is most commonly associated with a viral infection. The evidence of this association includes multiple antibodies for certain viruses, a prior upper respiratory tract infection, seasonal patterns, and case clusters (7). SAT has been linked with several respiratory viruses, including Epstein-Barr virus (8), coxsackievirus (9), and influenza virus (10). Nevertheless, there is currently no clear evidence as to decide if the follicular damage in SAT is caused by a direct viral infection of the gland or by an immune response to the virus (4). Angiotensin-converting enzyme 2 (ACE2), the receptor through which SARS-CoV-2 invades the cells, is expressed in many extrapulmonary tissues like thyroid follicular cells. Accordingly, the thyroid gland may be a potential target for viral damage because of its high ACE2 expression. COVID-19 can also affect thyroid function through other mechanisms, i. e, the autoimmune effects on the thyroid mediated by cytokine storm (11–13).

Because of the high susceptibility of thyroid involvement and inflammation during COVID-19, it is required to find out the relationship between these two disorders. As a result, the current systematic review targets to investigate and summarize the published articles exclusively focusing on COVID-19 infection-associated SAT.

## 2 Materials and methods

### 2.1 Search strategy

The present systematic review was performed pursuant to the Preferred Reporting Items for Systematic Reviews and Meta-Analysis (PRISMA) guidelines (14). The search strategy was developed using the Medical Subject Headings (MeSH) related terms/subheadings, including “Subacute Thyroiditis” and “COVID-19”. The following combination was searched for the subacute thyroiditis concept: “Subacute Thyroiditides” OR “Subacute Thyroiditis” OR “Subacute Painful Thyroiditis” OR “Painful Thyroiditis,” Subacute OR “Granulomatous Thyroiditides” OR “Subacute Nonsuppurative Thyroiditis” OR Thyroiditis, “Subacute Nonsuppurative” OR “De Quervain Thyroiditis” OR Thyroiditis, “De Quervain” OR “Giant Cell Thyroiditis” OR Thyroiditis, “Giant Cell.” Considering the concept of COVID-19, we searched the following combination: “COVID-19” OR COVID19 OR “SARS-CoV-2” OR “Coronavirus Disease-19” OR “SARS Coronavirus 2” OR “Coronavirus Disease 2019 Virus” OR “2019 Novel Coronavirus” OR “2019-nCoV” OR “Severe Acute Respiratory Syndrome Coronavirus 2”. The relevant studies published up to December 22, 2021, were searched through Scopus, PubMed/MEDLINE, Cochrane, Web of Science, ProQuest, and LitCovid databases. The search strategy was modified to fit each database. No restriction was imposed on the language or the time of publication. We updated the search on November 3, 2022, and extracted all the articles added to the above mentioned databases up to the stated date. Manual backward and forward citation searches were also conducted in order not to miss any of the publications.

### 2.2 Study selection

**Figure 1:**
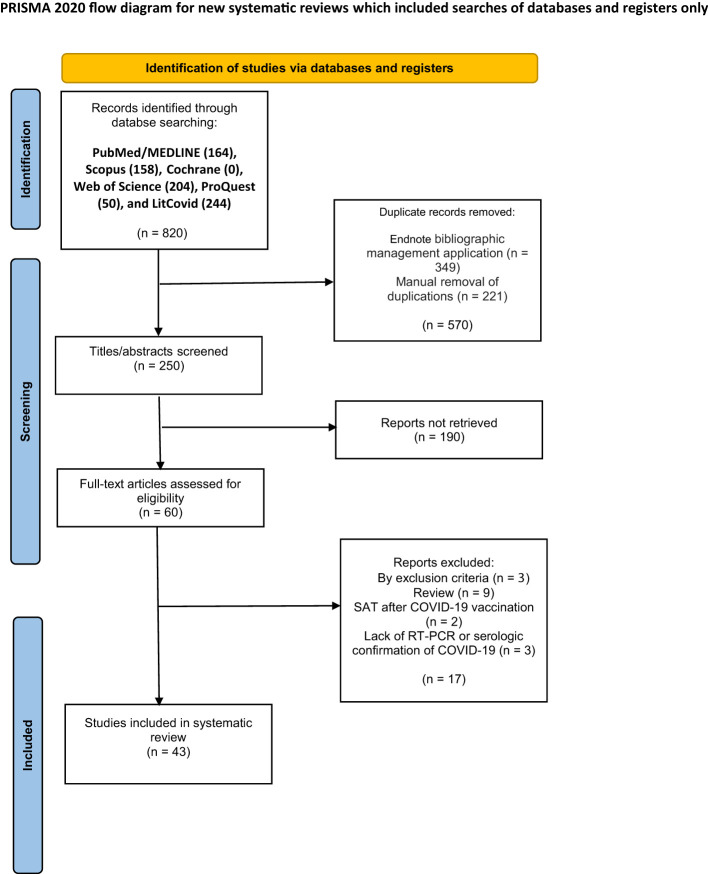
Preferred Reporting Items for Systematic reviews and Meta-Analyses (PRISMA) flow diagram of the study selection for the systematic review.

**Figure 1** depicts the completed search strategy results in the PRISMA flowchart. A total of 250 articles were retained after duplicate reports were removed by the Endnote bibliographic management application and then manually. The inclusion criteria of this systematic review are as follows:

1. The original articles describing the patients with confirmed SAT secondary to COVID-19.
2. Polymerase-Chain Reaction (PCR) and/or serologic confirmation of COVID-19.
3. SAT diagnosis by thyroid function tests and/or at least one imaging modality (thyroid ultrasound or radionuclide study) or cytology confirmation (fine needle aspiration).

Reviews, comments, expert opinions, studies reporting SARS-CoV-2 vaccine-associated SAT, and studies with SAT symptoms manifesting before COVID-19 were not included.

The titles and abstracts of the retained publications were independently screened by three authors (EM, KC, and SA) in two different phases to determine whether they met the requirements to be included. Following that, the authors (EM, KC, SK, RR, FZM, and SA) screened the full texts of the retained publications to assess their eligibility for inclusion.

### 2.3 Data extraction

The relevant information was extracted from the included publications and sorted into three categories:

1. The general information of the study: document type, the year of publication, the number of the study patients, authors, and the country of origin.
2. Patient data: the evaluation setting, gender, age, comorbidities, COVID-19 symptoms, and PCR or serology results for COVID-19. If the details of the COVID-19 symptoms were not reported, we only extracted the severity of COVID-19 (mild, moderate, or severe) as the classification by the World Health Organization required (15).
3. SAT Characteristics: the symptoms, physical examination findings, relevant laboratory tests, thyroid ultrasound, thyroid scan findings, fine needle aspiration (FNA) findings, the timing between respiratory and thyroid manifestations, management, outcome, and follow-up duration.

### 2.4 Quality assessment

Having extracted the above-mentioned data, the publications were screened regarding their quality of conduction and reporting. We used two relevant checklists, i. e., the Joanna Briggs Institute Critical Appraisal tools for use in Systematic Reviews—the Checklist for Case Reports and the Checklist for Case Series (16), based on which, the case reports and the case series were scored from zero to eight and ten, respectively. The quality of each publication was assessed by two authors individually, and a third author would decide on the score in case of disagreement.

## 3 Results

Forty-three articles [40 case reports (17–56) and three case series (57–59)] with 100 cases of SAT following COVID-19 were included in the present systematic review (**Figure 1**). The patients’ age ranged from 18 to 85 years (Mean: 42.70, SD: 11.85), and 68 (68%) of them were women. Comorbidities were reported in 10 patients, as stated in **Supplementary Table 1**. Eleven patients were reported as free from any comorbidities, and the presence or absence of comorbidities was not reported in the remaining 79 patients (79%). The cases were reported from Europe (36 cases, 36%), the Middle East (35 cases, 35%), Asia (21 cases, 21%), North America (seven cases, 7%), and South America (three cases, 3%). The details regarding the distribution of the countries are listed in **Supplementary Table 2**.

The time from the onset of COVID-19 to the onset of SAT symptoms varied from zero to 168 days (Mean: 28.31, SD: 36.92). In 9 patients (10%), COVID-19 and SAT were simultaneously diagnosed. Among the remaining 83 patients (89%), the mean duration between COVID-19 and SAT symptoms was reported as 33.51 days (SD: 33.69). The diagnosis was confirmed by SARS-CoV-2 PCR and the presence of IgG/IgM against SAR-COV-2 in 83 and 17 cases, respectively. In 65 patients, the following COVID-19 symptoms were detected: fever (27 cases, 36%), cough (20 cases, 27%), fatigue (14 cases, 19%), myalgia (12 cases, 16%), dyspnea (12 cases, 16%), anosmia (eight cases, 11%), sore throat (seven cases, 9%), headache (seven cases, 9%), and other less frequent symptoms as listed in **Supplementary Table 1**. The symptoms of 22 other patients were divided into three groups of mild (10 cases), moderate (seven cases), and severe COVID-19 (five cases). Ten patients (13%) had no symptoms of COVID-19, and the symptoms of COVID-19 were not reported in three patients.

Based on the data provided in **Supplementary Table 3**, the most prevalent symptoms in COVID-19-associated SAT were neck pain (69 cases, 69%), fever (54 cases, 54%), fatigue and weakness (34 cases, 34%), persistent palpitations (31 cases, 31%), tachycardia (15 cases, 15%), sweating (14 cases, 14%), and thyroid enlargement (14 cases, 14%). Mild to severe unilateral or bilateral neck pain radiating to the ear, jaw, or even the submandibular area was the main feature of the neck pain. Psychiatric symptoms such as agitation, irritability, anxiety, and mood changes were reported by three (3%), three (3%), one (1%), and one (1%) of the patients, respectively. Other less frequent symptoms were weight loss, tremor, myalgia, sore throat, cough, odynophagia, dysphagia, diarrhea, insomnia, chills, heat intolerance, and alopecia. The details of the symptoms are displayed in **Supplementary Table 1**. The symptoms of SAT were not mentioned in two patients (numbers 57 and 82). One of the patients out of 100 ones (number 40) had no symptoms associated with SAT. In this patient, the diagnosis was confirmed by the thyroid ultrasound and fine needle aspiration (FNA).

Heterogeneous findings were obtained from the thyroid ultrasonography on 92 patients. The results were as it follows: bilateral hypoechoic regions (61 cases, 66%), enlarged thyroid (46 cases, 50%), reduced vascularity (12 cases, 13%), isolated unilateral hypoechoic regions (12 cases, 13%), increased vascularity (two cases, 2%), and normal appearance (one case, 1%). In two (2%) of the 92 cases with reported ultrasonography results, it was only reported that the findings were “compatible with SAT” without any other details of the ultrasonography findings. Thyroid scintigraphy was performed in 34 cases (34%), demonstrating significantly-reduced or absent uptake in the thyroid gland in all these cases. In 15 cases (15%), the FNA of the thyroid gland was utilized in order to confirm the diagnosis.

As mentioned in **Supplementary Table 4**, the CRP level was measured in 87 patients and ranged from 1.05 mg/L to 347.22 mg/L (Mean: 64.00, SD: 71.49). The CRP was reported qualitatively “high” in five more patients. The CRP increased in 74 patients (85%), while ESR got elevated in 82 patients out of 83 ones (99%) with a reported ESR level and ranged from 18 to 140 mm/h (Mean: 65.87, SD: 28.30). In one patient with a normal ESR, the CRP was significantly increased. Thirteen individuals had normal white blood cell (WBC) count, 15 had raised WBC levels, and two had leukopenia. All patients had abnormal thyroid function tests at the onset of the disease, with TSH levels ranging from less than 0.001 to 1.53 mIU/L (Mean: 0.08, SD: 0.23), the FT4 levels from 0.96 to 7.7 ng/dl, and the FT3 levels from 2.07 to 21.6 ng/dL. Twenty-five patients had the reported serum thyroglobulin levels ranging from 2.4 to 187 ng/ml. Thyroglobulin was low in two patients who had auto-antibodies against thyroglobulin. Thyroid antibodies were reported as thyroid peroxidase antibody (anti-TPO) in 48, thyroglobulin antibody (anti-Tg) in 32, thyroid-stimulating immunoglobulin (TSI) in 16, and thyrotropin receptor antibody (TRAb) in 24 patients. Anti-TPO and anti-Tg were positive in eight (17%) and six patients (18%), respectively, with a maximum of 96.71 IU/ml and 512 IU/ml, respectively. TRAb and TSI results were all negative.

Thirty-one patients (31%) were admitted to the hospital, with 24 ones hospitalized because of SAT symptoms and six hospitalized due to COVID-19. Most of these cases were treated with prednisolone 20 to 50 mg daily (19 patients, 61%), aspirin combined with other drugs (three cases, 9%), aspirin alone (three cases, 9%), atenolol combined with corticosteroids (three cases, 9%), and dexamethasone 8 to 24 mg daily (three cases, 9%). Other prescribed drugs were methimazole (two cases, 6%), propranolol (one case, 3%), ibuprofen (one case, 3%), naproxen (one case, 3%), celecoxib (one case, 3%), and paracetamol (one case, 3%).

Sixty-eight patients (68%) were treated as outpatients, most of whom were treated with corticosteroids like prednisone and methylprednisolone (43 patients, 63%) or nonsteroidal anti-inflammatory drugs (NSAIDs) such as naproxen, ibuprofen, and aspirin (17 patients, 25%). The other medications utilized in the treatment of these patients were beta-blockers (seven cases, 10%), paracetamol (one case, 2%), colchicine (one case, 2%), and dexamethasone (one case, 2%). Two patients (3%) did not receive any treatment, and the treatment plan was not described for three other patients. The duration of the follow-up was reported in 86 patients and ranged from 4 days to 12 months (Mean: 61.59 days, SD: 67.07). The follow-ups included evaluating the clinical symptoms, imaging, and laboratory studies. The findings of the follow-ups were reported in 90 patients. Of twenty-one patients (23%) who developed hypothyroidism in the follow-up, six ones (6% of the total population) were treated with levothyroxine and the rest of the patients received no medication for hypothyroidism.

## 4 Discussion

Thyroid dysfunction has been reported in numerous patients during the COVID-19 pandemic, indicating that SARS-CoV-2 may have played a role in thyroid damage either during or after the infection. Compared to other subtypes of thyroiditis, SAT has been more commonly linked to viral infections, particularly those of the upper respiratory tract, such as coronaviruses (11). Thyrotoxicosis, hypothyroidism, and sick euthyroid syndrome are three common thyroid complications among the effects of SARS-CoV-2 on the thyroid gland (12). Consequently, predicting thyroid function in COVID-19 patients is challenging.

SARS-CoV-2 has the potential to destroy the thyroid tissue directly or indirectly. The most accepted mechanism involves direct viral damage through transmembrane serine protease 2 and angiotensin-converting enzyme 2 receptors on the surface of the thyroid follicular cells (60, 61). In addition to the mentioned mechanism, other mechanisms, such as indirect immune-mediated responses and medicines prescribed for COVID-19, are also thought to be responsible for thyroid damage (11–13).

Prior viral infection is considered a risk factor in genetically susceptible individuals. As argued by Stasiak et al., the presence of either HLA-B*35 or HLA-C*04:01 alleles or both are genetic markers of susceptibility to SAT. As this theory states, HLA-mediated tissue destruction occurs through either cross-reaction with host antigens or auto-antibody formation against the large amount of self-antigens released from the damaged thyroid tissue. Although no HLA testing was performed on any of the cases we reviewed, Stasiak et al. suggest that the presence of novel high-risk alleles, including HLA-B*18:01 and HLA-DRB1*01, may deteriorate the clinical course of SAT (62, 63). Another hypothesis involves molecular mimicry; a recent *in-vitro* investigation revealed that antibodies against SARS-CoV-2 antigens may cross-react with self-tissue antigens, such as TPO. This mechanism is a putative pathogenic trigger for immune-mediated adverse effects following SARS-CoV-2 infection and immunization (63).

Similar to classic SAT and prior reviews on COVID-19-associated SAT (12), the current systematic review study was performed on the patients aged 18 to 85 years, with a majority of women (68%). The age range in the present study was longer than that of several previously-published reviews (4, 11, 64–66). In line with the most prevalent COVID-19 symptoms (66, 67), fever and cough were the two most frequent complaints of the patients during COVID-19 infection and before SAT manifestations. Moreover, the asymptomatic patients and those with severe COVID-19 symptoms, as defined by the WHO (15), had similar SAT presentations.

In the present study, the time from the beginning of COVID-19 infection to the onset of SAT ranged from zero to 168 days, which is longer than that of previous studies (4, 11, 59, 65, 66). There were two patients with a lag of 168 days between COVID-19 diagnosis and SAT manifestation. Although the relatively prolonged lag may question the correlation between these two entities, we included these two cases based on their positive serology (IgM, IgG) when SAT manifested (58). Similar to classic SAT and prior reviews (4, 11, 64–66), the most prevalent symptom was neck pain (69%) in the present study. Fever (54%) was the second most common symptom in the present study, congruent with merely two of the previously conducted studies (11, 66). The early cases of COVID-19-associated SAT generally reported suffering from severe neck discomfort. In contrast, the authors of observational studies of COVID-19 patients in intensive care units described uncommon cases of painless SAT with no local symptoms (30, 59). Although we discovered heterogeneity among the inpatient cases, it is proposed that insufficient lymphocytic infiltration within the thyroid gland due to COVID-19-associated lymphopenia results in the absence of tension of the thyroid capsule and, thus, painless presentations of thyroiditis (59). Furthermore, the growing evidence supports the complicated connection between thyroid hormones and CNS neurotransmitters, which has the potential to bring about neuropsychiatric symptoms and raise patient risks (68, 69). Nevertheless, the outcomes in our cases with the stated disorders were similar, and the treatment was successful.

Consistent with early reviews (4, 11, 64–66), we observed at least one of the ESR or CRP variables elevating in all patients. The ESR increased in the majority of the cases (99%), while the CRP increased in 85%. The mean ESR observed in our study was 65.87 mm/hr; however, ESR in SAT might increase up to three-digit values (70). Additionally, nearly 60% had low TSH levels during the early phase of the illness, with high free T3 and free T4 levels. Following the classic SAT (11, 25, 71), 13 individuals had normal WBC levels, 15 cases had elevated WBC levels, as being also indicated in a few patients in another study (65), while two revealed leukopenia in contrast.

The clinical SAT symptoms might not be typical; thus, thyroid ultrasonography may help to get to the correct diagnosis. In this regard and in agreement with almost all the previous cases (4, 11, 64–66), 92 patients in this review had the classic sonographic findings of SAT, which include bilateral or unilateral, localized or multifocal, and poorly defined hypoechoic regions (72). The previously conducted studies also found that in order to assess thyroid function, radionuclide scanning combined with ultrasound examination could significantly increase the diagnostic rate of SAT. In the mentioned instances, radionuclide uptake in the thyroid gland ranges from very low or absent in the hyperthyroid phase to a normal appearance in the late recovery phase (73, 74). Confirming the findings of the previous studies (4, 64, 65), there was a significant decrease in radionuclide uptake in all 34 cases undergoing thyroid scintigraphy. A previously firm thyroid nodule can rapidly grow in SAT setting, initially suggesting thyroid malignancy (70). Thus, the clinicians are obliged to be aware of this matter that SAT might present as suspicious nodular lesions on ultrasound and may require thyroid FNA, which is rarely used in diagnostic workups. However, one study suggested that margin, vascularity, and echogenicity may help differentiate the characteristics of SAT and malignancy and thus, the requirement for FNA gets reduced (75). Fifteen of the patients we reviewed underwent an FNA to confirm the diagnosis.

The serologic evaluations from previous studies frequently reveal normal thyroid antibody levels in cases with classic SAT. In fact, some trials reported positive transient results of anti-TPO, TSI, anti-Tg, and TRAb in some non-autoimmune thyroid diseases, including SAT, suggesting that immunological hyperactivity in COVID-19 might have led to the formation of these antibodies (4, 12). In our study, TRAb and TSI were negative in all 24 and 16 cases being screened in this respect. However, anti-TPO was assessed in 48 cases and was found positive in eight patients. Moreover, out of 32 individuals who had anti-Tg results, six were positive. Two of these patients with positive anti-Tg had normal thyroglobulin levels, possibly due to the presence of this autoantibody and its effect on thyroglobulin (76). Similarly, some relevant reviews reported the new detection of autoantibodies in SAT patients (4, 11, 64, 65). Based on our findings and consistent with a previous study (4), the patients with positive and negative auto-antibodies received various treatments, and all recovered comparably.

Thirty-one patients had to be hospitalized due to their risk factors or the severity of their symptoms. However, their medical procedures and results were comparable to those of the patients with less severe conditions. Therefore, NSAIDs, beta-blockers, and glucocorticoids may occasionally be advised due to the self-limiting nature of SAT (11). In this respect, 87 patients of 100 cases (87%) in our review received either of these three categories of drugs. Sixty-six received glucocorticoids, 26 NSAIDs, 11 beta-blockers, two methimazole, and one colchicine. For three patients, the treatment was not reported, and two patients did not receive any treatment. Based on the literature, glucocorticoids (4, 11, 64–66) are the preferred treatment for SAT secondary to COVID-19, and beta-blockers (4, 11, 64, 65) and NSAIDs (4, 64) are other prescribed medications, respectively.

The three clinical manifestations of SAT are hyperthyroidism, transient hypothyroidism, and normal thyroid function (13). SAT patients often require three months to return to a normal euthyroid condition (25, 59). Nevertheless, with the available medications, the duration from the symptom improvement to complete recovery ranges from two days to three months. Moreover, only about one-fourth of our patients showed temporary hypothyroidism until the end of their follow-up. Previous reviews with smaller sample sizes (4, 11, 64–66) revealed 10% to 36.5% hypothyroidism incidence during follow-up. Thus, about 10% of the individuals could develop persistent hypothyroidism (12), which was not reported in the studied cases of the present study. Besides, a follow-up period of up to twelve months was taken into account to prevent the potential adverse outcomes. However, no serious adverse event was reported.

## 5 Strengths and limitations

The present study reviewed the current literature systematically and comprehensively regarding the characteristics and manifestations of subacute thyroiditis in COVID-19. However, this study had its own limitations. The case reports and the case series constituted the majority of the included articles, affecting the quality of the results in our study. Additionally, some variables of interest were not reported in the included studies, which limited an otherwise more comprehensive review.

## 6 Conclusion

We reviewed all the relevant cases with the required details—including history, physical examination, lab tests, and imaging—to be able to ascertain the earlier relevant findings. Although we found no significant clinical differences between the typical and post-COVID-19 SAT, the immunological, viral, or pharmaceutical reactions may cause minor discrepancies between the classic and COVID-19-associated SAT. The literature is controversial on how post-viral SAT symptoms, thyroid function problems, and medications may affect the findings.

## Data availability statement

The original contributions presented in the study are included in the article/**Supplementary Material**. Further inquiries can be directed to the corresponding authors.

## Author contributions

EM and KC-Z designed the study. EM performed the bibliographic search, prepared the tables, and critically revised the manuscript. EM, SK, RR, FZM, KC-Z, and SA screened the articles, extracted the data, and assessed the quality of the included articles. KC-Z, SK, RR, and FZM wrote the initial version of the manuscript. All authors contributed to the article and approved the submitted version.
