# Supplementary material for: Subacute thyroiditis following COVID-19: A systematic review
Source: Front Endocrinol (Lausanne). 2023 Apr 5;14:1126637. doi: 10.3389/fendo.2023.1126637 (PMC10115182; doi:10.3389/fendo.2023.1126637)
Supplement: Supplementary file 1 [file DataSheet_1.pdf]

Supplementary Material

Supplementary Table 1. Symptoms and comorbidities of the patients

Variable	Number of patients (%)
**COVID-19 symptoms**	
**Fever**	27 (36)
**Cough**	20 (27)
**Fatigue or weakness**	14 (19)
**Myalgia**	12 (16)
**Dyspnea**	12 (16)
**Asymptomatic**	10 (13)
**Anosmia**	8 (11)
**Sore throat**	7 (9)

**Headache**	7 (9)
**Rhinorrhea**	4 (5)
**Diarrhea**	4 (5)
**Coryza**	4 (5)
**Odynophagia or dysphagia**	4 (5)
**Anorexia**	2 (3)
**Tachycardia**	2 (3)
**Undefined upper respiratory symptoms**	2 (2)
**Chest discomfort**	2 (2)
**Flu-like symptoms**	1 (1)
**Hypoxia**	1 (1)
**Tachypnea**	1 (1)
**Weight loss**	1 (1)

**Sleepiness**	1 (1)
**Apathy**	1 (1)
**Subacute thyroiditis symptoms**	
**Neck pain**	69 (69)
**Fever**	54 (54)
**Fatigue or weakness**	34 (34)
**Persistent palpitations**	31 (31)
**Tachycardia**	15 (15)
**Sweating**	14 (14)
**Thyroid enlargement**	14 (14)
**Weight loss**	8 (8)
**Tremor**	8 (8)
**Myalgia**	7 (7)
**Sore throat**	6 (6)

**Cough**	5 (5)
**Odynophagia**	5 (5)
**Diarrhea**	4 (4)
**Insomnia**	4 (4)
**Dysphagia**	3 (3)
**Chills**	3 (3)
**Heat intolerance**	3 (3)
**Irritability**	3 (3)
**Agitation**	3 (3)
**Anxiety**	1 (1)
**Mood changes**	1 (1)
**Alopecia**	1 (1)
**Staring**	1 (1)
**Delirium**	1 (1)

**Feeling of suffocation**	1 (1)
**Abdominal pain**	1 (1)
**Back pain**	1 (1)
**Hot flashes**	1 (1)
**Asymptomatic**	1 (1)
**Comorbidities**	
**Not reported**	79 (79)
**None**	11 (11)
**Hypertension**	4 (4)
**Diabetes**	3 (3)
**Anxiety**	2 (2)
**Chronic kidney disease**	2 (2)
**Depression**	2 (2)
**Non-toxic nodular goiter**	1 (1)

**Anemia**	1 (1)
**Atrial fibrillation**	1 (1)
**Coronary artery disease**	1 (1)
**Treated supraventricular tachycardia**	1 (1)
**Treated scoliosis**	1 (1)
**Asthma**	1 (1)
**Gastroesophageal reflux disease**	1 (1)
**Glomerulonephritis**	1 (1)
**Breast cancer**	1 (1)

Supplementary Table 2. Baseline and COVID-19 characteristics of the patients

Reference	Case Number	Author	Country	Document type	Number of patients	Quality assessment score	Setting	Gender	Age (years)	Comorbidities	Symptoms of COVID-19	PCR
**(1)**	1	Feghali et al.	USA	Case report	1	3	Outpatient	Female	41	NR	Headache, fatigue, anorexia, and mild fever	Positive
**(2)**	2	Sohrabpour et al.	Iran	Case report	6	5	Outpatient	Female	26	NR	Dry cough	Negative, but Positive Serology (IgM, IgG)
**(2)**	3	Sohrabpour et al.	___	___	___	___	Outpatient	Female	37	NR	Myalgia	Negative, but Positive Serology (IgM, IgG)
**(2)**	4	Sohrabpour et al.	___	___	___	___	Outpatient	Male	35	NR	Asymptomatic	Negative, but Positive Serology (IgM, IgG)
**(2)**	5	Sohrabpour et al.	___	___	___	___	Outpatient	Female	41	NR	Mild fever and myalgia	Negative, but Positive Serology (IgM, IgG)
**(2)**	6	Sohrabpour et al.	___	___	___	___	Outpatient	Male	52	NR	Dry cough, mild myalgia, and fever	Negative, but Positive Serology (IgM, IgG)
**(2)**	7	Sohrabpour et al.	___	___	___	___	Outpatient	Female	34	NR	Asymptomatic	Negative, but Positive Serology (IgM, IgG)
**(3)**	8	Osorio et al.	Mexico	Case report	1	8	Inpatient	Male	64	None	High fever, productive cough, headache, and extreme fatigue	Positive

**(4)**	9	Ippolito et al.	Italy	Case report	1	7	Inpatient	Female	69	Recent back surgery, non-toxic nodular goiter with a dominant euthyroid right lobe nodule	Mild fever, cough, and dyspnea	Positive
**(5)**	10	Ünübol et al.	Turkey	Case report	2	6	Outpatient	Male	56	NR	Mild fever, cough, and myalgia	Positive
**(5)**	11	Ünübol et al.	___	___	___	___	Outpatient	Female	38	NR	Sore throat	Positive
**(6)**	12	Ayhan and Guner	Turkey	Case report	1	7	Outpatient	Male	39	None	Sore throat, fatigue, and fever	Positive
**(7)**	13-30	Brancatella et al.	Italy	Case series	18	8	Outpatient	Female	34 (±14)	NR	NR	Positive
**(8)**	31	de la Higuera Lopez-Frias et al.	Spain	Case report	1	7	Outpatient	Female	36	None	Fever and myalgia	NR, but Positive Serology (IgM, IgG)
**(9)**	32	Khatri et al.	USA	Case report	1	7	Inpatient	Female	41	Supraventricular tachycardia treated with ablation, anxiety, depression, treated Helicobacter pylori infection, anemia, surgically corrected scoliosis, and gastroesophageal reflux disease	Fever, cough, and coryza	Positive
**(10)**	33	Ruano et al.	Spain	Case report	1	7	Inpatient	Female	28	None	Diarrhea and abdominal pain	Positive
**(11)**	34	Baykan and Carlioglu	Turkey	Case report	5	5	Outpatient	Female	51	NR	Odynophagia, myalgia, and anosmia	Positive
**(11)**	35	Baykan and Carlioglu	___	___	___	___	Outpatient	Male	39	NR	Mild fever, cough, and dyspnea	Positive

**(11)**	36	Baykan and Carlioglu	___	___	___	___	Outpatient	Female	31	NR	Mild upper respiratory symptoms	Positive
**(11)**	37	Baykan and Carlioglu	___	___	___	___	Outpatient	Female	27	NR	Myalgia, weakness, and fever	Positive
**(11)**	38	Baykan and Carlioglu	___	___	___	___	Outpatient	Male	47	NR	Mild fever, myalgia, and cough	Positive
**(12)**	39	Abreu et al.	Brazil	Case report	3	6	Outpatient	Female	34	Breast cancer	Dyspnea	Positive
**(12)**	40	Abreu et al.	___	___	___	___	Outpatient	Female	34	NR	Dyspnea	Positive
**(12)**	41	Abreu et al.	___	___	___	___	Outpatient	Female	39	NR	Mild fever and anosmia	Positive
**(13)**	42	Mathews et al.	USA	Case report	1	7	Inpatient	Male	67	Heart failure, coronary artery disease, diabetes mellitus type 2, chronic kidney disease, and atrial fibrillation	Shortness of breath and diarrhea	Positive
**(14)**	43	Brancatella et al.	Italy	Case report	1	6	Outpatient	Female	18	NR	Rhinorrhea and cough	Positive
**(15)**	44	Chakraborty et al.	India	Case report	1	7	Outpatient	Male	58	Diabetes	Sore throat and mild fever	Positive
**(16)**	45	Campos-Barrera et al.	Mexico	Case report	1	6	Outpatient	Female	37	None	Odynophagia and anosmia	Positive
**(17)**	46	Mattar et al.	Singapore	Case report	1	7	Inpatient	Male	34	NR	Fever, dry cough, headache, and anosmia	Positive

**(18)**	47	Timofeeva et al.	Russia	Case report	1	7	Outpatient	Male	56	NR	Dry cough, fever, dyspnea, apathy, and fatigue	Positive
**(19)**	48	Guven	Turkey	Case report	1	7	Outpatient	Male	49	None	Cough and shortness of breath	Positive
**(20)**	49	Chong et al.	USA	Case report	1	6	Outpatient	Male	37	NR	Cough, fever, chills, and dyspnea	Positive
**(21)**	50	Semikov et al.	Russia	Case report	2	6	Outpatient	Female	45	NR	Nasal congestion, cough, weakness, muscle pain, and headache	Positive
**(21)**	51	Semikov et al.	___	___	___	___	Outpatient	Female	40	NR	Mild symptoms	Positive
**(22)**	52	Kong and La Porteb	UK	Case report	1	7	Outpatient	Male	44	Hypertension, chronic glomerulonephritis, and stage III chronic kidney disease	Shortness of breath, loose stools, fatigue, and hypoxia	Positive
**(23)**	53	Tjønnfjord et al.	Norway	Case report	1	7	Inpatient	Male	in his forties	NR	Mild fever, cough, and dyspnea	Positive
**(24)**	54	Álvarez Martín et al.	Spain	Case report	1	7	Outpatient	Female	46	NR	Asymptomatic	Negative, but Positive Serology
**(25)**	55	Davoodi et al.	Iran	Case report	1	6	Inpatient	Male	33	NR	Fever, sore throat, myalgia, and lethargy	Positive
**(26)**	56	Stasiak et al.	Poland	Case report	4	6	Outpatient	Male	50	NR	Cough, fever, headache, malaise, anosmia, chest discomfort	Positive
**(26)**	57	Stasiak et al.	___	___	___	___	Outpatient	Female	39	NR	Fever, cough, headache, and malaise	Positive

**(26)**	58	Stasiak et al.	___	___	___	___	Outpatient	Female	55	NR	Moderate symptoms	Positive
**(26)**	59	Stasiak et al.	___	___	___	___	Outpatient	Female	57	NR	Mild symptoms	Positive
**(27)**	60	Brancatella et al.	Italy	Case report	3	7	Outpatient	Female	38	NR	Respiratory symptoms	Positive
**(27)**	61	Brancatella et al.	___	___	___	___	Outpatient	Female	29	NR	Rhinorrhea	Positive
**(27)**	62	Brancatella et al.	___	___	___	___	Outpatient	Female	46	NR	Fever, cough, rhinorrhea, anosmia, asthenia, and weight loss	Positive
**(28)**	63	Sato et al.	Japan	Case report	1	7	Outpatient	Female	31	NR	Mild fever and sore throat	Positive
**(29)**	64	San Juan et al.	Philippine s	Case report	1	6	Inpatient	Female	47	Asthma	NR	Positive
**(30)**	65	Ruggeri et al.	Greece	Case report	1	7	Inpatient	Female	43	NR	Fever, rhinorrhea, odynophagia, cough, hoarseness, and conjunctivitis	Positive
**(31)**	66	Mehmood et al.	Pakistan	Case report	1	7	Inpatient	Female	29	None	NR	Negative, but Positive Serology (IgM, IgG)
**(32)**	67	Seyed Resuli and Bezgal	Turkey	Case report	5	5	Inpatient	Female	32	NR	Asymptomatic	Positive
**(32)**	68	Seyed Resuli and Bezgal	___	___	___	___	Inpatient	Female	25	NR	Asymptomatic	Positive
**(32)**	69	Seyed Resuli and Bezgal	___	___	___	___	Inpatient	Female	45	NR	Asymptomatic	Positive

**(32)**	70	Seyed Resuli and Bezgal	___	___	___	___	Inpatient	Female	29	NR	Asymptomatic	Positive
**(32)**	71	Seyed Resuli and Bezgal	___	___	___	___	Inpatient	Female	21	NR	Asymptomatic	Positive
**(33)**	72	Bahçecioğlu et al.	Turkey	Case series	9	8	Outpatient	Male	40	NR	Mild symptoms	Negative, but Positive Serology (IgM, IgG)
**(33)**	73	Bahçecioğlu et al.	___	___	___	___	Outpatient	Female	54	NR	Mild symptoms	Negative, but Positive Serology (IgM, IgG)
**(33)**	74	Bahçecioğlu et al.	___	___	___	___	Outpatient	Male	52	NR	Mild symptoms	Negative, but Positive Serology (IgM, IgG)
**(33)**	75	Bahçecioğlu et al.	___	___	___	___	Outpatient	Male	37	NR	Mild symptoms	Negative, but Positive Serology (IgM, IgG)
**(33)**	76	Bahçecioğlu et al.	___	___	___	___	Outpatient	Male	51	NR	Mild symptoms	Negative, but Positive Serology (IgM, IgG)
**(33)**	77	Bahçecioğlu et al.	___	___	___	___	Outpatient	Female	36	NR	Mild symptoms	Negative, but Positive Serology (IgM, IgG)
**(33)**	78	Bahçecioğlu et al.	___	___	___	___	Outpatient	Female	53	NR	Mild symptoms	Positive
**(33)**	79	Bahçecioğlu et al.	___	___	___	___	Outpatient	Male	50	NR	Mild symptoms	Positive
**(33)**	80	Bahçecioğlu et al.	___	___	___	___	Outpatient	Female	48	NR	Moderate symptoms	Negative, but Positive Serology (IgM, IgG)
**(34)**	81	Elawady et al.	Egypt	Case report	1	6	Outpatient	Female	33	Depression, anxiety, and pregnancy induced hypertension	Flu-like symptoms	Positive

**(35)**	82	Jakovac et al.	Croatia	Case report	1	2	Inpatient	Male	85	NR	Tachypnea, tachycardia, hypotension, and cyanosis	Positive
**(36)**	83	Salehi et al.	Iran	Case report	1	7	Outpatient	Male	55	None	Cough	Positive
**(37)**	84	Hajósi-Kalcakosz et al.	Budapest	Case report	1	7	NA	Female	31	None	Fever, headache, myalgia, sleepiness, anosmia, and ageusia	Positive
**(38)**	85	Ashraf et al.	Pakistan	Case report	1	7	Inpatient	Male	58	Diabetes and hypertension	Fever, myalgia, and fatigue	Positive
**(39)**	86-96	Mondal et al.	India	Case series	11	8	Inpatient	Mainly female (n=7, 63.63%)	Mean (SD): 44.09 (16.62)	NR	One asymptomatic, five moderate, and five severe symptoms of COVID-19	Positive
**(40)**	97	Kleim et al.	Germany	Case report	1	6	Inpatient	Male	44	NR	Chills and physical exhaustion	Positive
**(41)**	98	Al-Shammaa and Abdlkadir	Iraq	Case report	1	6	Outpatient	Female	53	None	Fever and sore throat	Positive
**(42)**	99	Dolkar et al.	USA	Case report	1	4	Inpatient	Female	55	Hypertension	Respiratory failure	Positive
**(43)**	100	Poudel Jaishi et al.	Nepal	Case report	1	5	Outpatient	Male	45	None	Dyspnea, sweating, fatigue, sore throat, dysphagia, diarrhea, chest compression, anorexia, and tachycardia (undistinguishable from COVID-19 symptoms, because they coincided)	NR, but Positive Serology (IgM, IgG)

NR: not reported; SD: standard deviation; n: number.

Supplementary Table 3. Subacute thyroiditis manifestations and management

Reference	Case Num-ber	Author	Thyroiditis symptoms	Thyroid ultrasound	Gap between respiratory and thyroid manifestations	Management	Follow-up Duration	Outcome
**(1)**	1	Feghali et al.	Persistent palpitations and insomnia	NR	6 weeks	Initial management NR, levothyroxine 112 µg daily for the hypothyroid phase	3 weeks	With a TSH 67.04 mIU/L and free T4 0.4 ng/dl after three weeks, levothyroxine 112 µg/day was started (final outcome NR).
**(2)**	2	Sohrabpour et al.	High-grade fever, fatigue, palpitations, markedly painful, tender, and slightly enlarged thyroid gland	Bilateral hypoechoic areas	NR	Prednisolone 25 mg/day for 10 days, tapered in 4 weeks	1 month	Symptomatic relief after a week. After a month, laboratory test results were as follows: ESR 17, TSH 2.21, FT4 17.9.
**(2)**	3	Sohrabpour et al.	High-grade fever, fatigue, palpitations, markedly painful, tender, and slightly enlarged thyroid gland	Bilateral hypoechoic areas	NR	Prednisolone 25 mg/day for 10 days, tapered in 4 weeks	1 month	Symptomatic relief after a week. After a month, laboratory test results were as follows: ESR 4, TSH 1.83, FT4 19.3.
**(2)**	4	Sohrabpour et al.	High-grade fever, fatigue, palpitations, markedly painful, tender, and slightly enlarged thyroid gland	Bilateral hypoechoic areas	NR	Prednisolone 25 mg/day for 10 days, tapered in 4 weeks	1 month	Symptomatic relief after a week. After a month, laboratory test results were as follows: ESR 9, TSH 3.75, FT4 13.2.
**(2)**	5	Sohrabpour et al.	High-grade fever, fatigue, palpitations, markedly painful, tender, and slightly enlarged thyroid gland	Bilateral hypoechoic areas	NR	Prednisolone 25 mg/day for 10 days, tapered in 4 weeks	1 month	Symptomatic relief after a week. After a month, laboratory test results were as follows: ESR 25, TSH 1.84, FT4 18.9.
**(2)**	6	Sohrabpour et al.	High-grade fever, fatigue, palpitations, markedly painful, tender, and slightly enlarged thyroid gland	Bilateral hypoechoic areas	NR	Prednisolone 25 mg/day for 10 days, tapered in 4 weeks	1 month	Symptomatic relief after a week. After a month, laboratory test results were as follows: ESR 28, TSH 0.46, FT4 20.2.

**(2)**	7	Sohrabpour et al.	High-grade fever, fatigue, palpitations, markedly painful, tender, and slightly enlarged thyroid gland	Bilateral hypoechoic areas	NR	Prednisolone 25 mg/day for 10 days, tapered in 4 weeks	1 month	Symptomatic relief after a week. After a month, laboratory test results were as follows: ESR 2, TSH 3.67, FT4 12.7.
**(3)**	8	Osorio et al.	Slight distal tremor with evident generalized diaphoresis and impalpable thyroid gland	Diffusely enlarged micro-nodular thyroid gland	3 weeks	Atenolol 50 mg twice daily and prednisone 50 mg daily with tapering in 2 weeks	NR	Symptomatic relief after three days. After detecting a primary hypothyroidism in the follow-up, levothyroxine 100 µg/day was started (the timing and final outcome: NR).
**(4)**	9	Ippolito et al.	Palpitations, insomnia, and agitation	Enlarged hypoechoic thyroid, decreased vascularity and a known 30-mm homogeneous nodule in the right lobe (with peripheral vascularization)	5 days	Methylprednisolone 40 mg intravenous for 3 days, then continuing with 25 mg/day oral prednisone, tapered in 4 weeks or more, according to clinical response.	10 days	Ten days after starting steroids, FT4 was 21.9 pg/ml and FT3 was 3.07 pg/ml.
**(5)**	10	Ünübol et al.	Severe pain, tenderness, and swelling of thyroid radiating to the right ear, fatigue, and myalgia	Hypoechoic, hypovascular areas of the right lobe, focally heterogeneous left lobe	4 weeks	Oral naproxen 550 mg twice a day for four weeks	4 weeks	Pain and full symptomatic relief within two and ten days, respectively. TFT and inflammatory markers were normal after four weeks of treatment.
**(5)**	11	Ünübol et al.	Anterior neck pain, myalgia, fatigue, back pain, headache, and a tender and large thyroid gland	Bilateral and diffuse hypoechoic and hypovascular areas	5 weeks	Oral naproxen 550 mg twice a day for four weeks	4 weeks	Pain relief in two days. TFT and inflammatory markers were normal after four weeks of treatment, although ESR was still elevated after four weeks (=23).
**(6)**	12	Ayhan and Guner	Sinus tachycardia, pain, swelling, and tenderness in the anterior neck, fatigue, myalgia, palpitation, and tremor	Compatible with subacute thyroiditis (no findings were described)	2 weeks	Prednisolone 16 mg/day with taper after one week, ibuprofen 1200 mg/day, and propranolol 20 mg three times a day	5 weeks	Complete recovery in five weeks
**(7)**	13-30	Brancatella et al.	Bilateral neck pain in 16 (89%) and Fever in 17 (94%)	Thyroid volume (IQR): 22 (5)	Median (IQR): 29 (12)	Steroids in 16, NSAID in 1, and None in 1. Median duration (days) of steroid treatment was 85 (IQR=13).	Median: 3 months	Hypothyroidism in 13 of 15 patients (%87) with a follow-up of 3 months. Nine of these patients were checked at 6 months and were all still hypothyroid.

**(8)**	31	de la Higuera Lopez-Frias et al.	Anterior neck tenderness and pain radiating to the jaw, mild fever, palpitations, and fatigue	Compatible with subacute thyroiditis (no findings were described)	About 45 days	Ibuprofen 600 mg/day and propranolol 10 mg/day	5 months	Symptomatic relief and decrease of inflammatory markers after 15 days, normalization of TFT and inflammatory markers after five months
**(9)**	32	Khatri et al.	Anterior neck pain radiating to the jaw, odynophagia, fever, chills, diaphoresis, weight loss, fatigue, alopecia, heat intolerance, irritability, headache, tremor, and palpitations	Heterogeneous thyroid gland with bilateral patchy ill-defined hypoechoic areas	4 weeks	Ibuprofen 600 mg/6 h and prednisone 40 mg/day, with a 4-week corticosteroid taper	45 days	Symptomatic relief after 2 days
**(10)**	33	Ruano et al.	Fever, neck pain radiating to the jaw, sore throat, palpitations, severe asthenia, sinus tachycardia, and a grade 2 goiter	NR	About 1 month	Paracetamol 1 g/8 h for 10 days, then 500 mg of aspirin and 40 mg of propranolol, both every 6 h	2 weeks	Symptomatic relief in 24 hours after the initiation of aspirin, with a complete recovery in 2 weeks
**(11)**	34	Baykan and Carlioglu	Fatigue and neck pain radiating to the right jaw and ear	Widely enlarged and hypoechogenic thyroid gland	18 days	NSAID for ten days, then oral prednisolone 40 mg/day with gradual tapering of the dose	6 weeks	Rapid clinical relief after corticosteroid therapy and normal TFT after six weeks
**(11)**	35	Baykan and Carlioglu	Palpitations, tachycardia, sweating, and neck pain	Diffuse hypoechoic areas	22 days	NSAID and oral prednisolone treatment for 4 weeks	4 weeks	Clinical and TFT normalization after four weeks of corticosteroid therapy
**(11)**	36	Baykan and Carlioglu	Palpitations, fever, fatigue, and neck pain spreading to the right jaw	Widely enlarged and hypoechogenic thyroid gland	25 days	NSAID for a week, then oral prednisolone 40 mg/day for 5 weeks with tapering	5 weeks	Rapid clinical relief after corticosteroid therapy and normal TFT after five weeks
**(11)**	37	Baykan and Carlioglu	Fatigue and anterior neck pain	Multiple hypoechoic areas	20 days	NSAID, then oral steroid treatment for 5 weeks	5 weeks	Clinical and TFT normalization after five weeks of corticosteroid therapy
**(11)**	38	Baykan and Carlioglu	Tachycardia, mild fever, and neck pain	Widely enlarged and hypoechoic thyroid gland	24 days	NSAID then steroids	NR	Symptomatic relief after corticosteroid therapy
**(12)**	39	Abreu et al.	Mild pain in the anterior cervical region and a mild fever	Hypoechoic area in the right thyroid lobe	28 days	Prednisone 15 mg/day	4 days	Symptomatic relief within four days

**(12)**	40	Abreu et al.	Asymptomatic	Hypoechoic area in the right thyroid lobe, 10 days after diagnosis	10 days	Prednisone	18 days	TFT normalization within 18 days
**(12)**	41	Abreu et al.	Pain in the anterior cervical region	A solid hypoechoic nodule in the left thyroid lobe	26 days	NR	2 weeks	Normal ultrasound after two weeks
**(13)**	42	Mathews et al.	Weight loss, fatigue, and diarrhea	Mildly enlarged thyroid gland and 5-mm bilateral cysts	Simultaneously	Methimazole 20 mg/day, then prednisone with tapering	About a year	Free T4 was 1.36 ng/dL and TSH was 0.007 uIU/mL within a week. TFT was normal at the last follow-up.
**(14)**	43	Brancatella et al.	Fever, neck pain radiating to the jaw, and palpitations	Bilateral and diffuse hypoechoic areas	15 days	Prednisone 25 mg/day	6 weeks	Neck pain and fever resolved after 2 days and the remaining symptoms within 1 week. TFT and CRP were normal at the last follow-up, and ESR was slightly elevated.
**(15)**	44	Chakraborty et al.	Neck pain, high grade fever, tachycardia, increased frequency of stools, and diffuse thyroid enlargement	Diffuse bilateral thyroid enlargement with hypoechogenicity, increased vascularity, and a solitary nodule in each lobe	Simultaneously	Propranolol 40 mg/day and oral prednisolone 30 mg/day with tapering over one month	1 month	Hypothyroidism after a month that was managed with oral levothyroxine (50 µg/day) with periodic monitoring of TFT.
**(16)**	45	Campos-Barrera et al.	Fatigue and neck pain irradiating to the right jaw and ear	NR	1 month	NR	1 month	Clinical relief after a month, in spite of altered ESR and TSH (0.01 mUI/L)
**(17)**	46	Mattar et al.	Diffuse thyromegaly, anterior neck pain refractory to symptomatic treatment, and a new-onset tachycardia	Thyromegaly, bilateral hypoechoic areas with ill-defined margins, and bilateral hypovascularity	9 days	Prednisolone 20 mg/day and Atenolol 25 mg/day	10 weeks	Symptomatic relief after two days and significant decrease in CRP after five days
**(18)**	47	Timofeeva et al.	Feeling of suffocation, weakness, pain and pressure in the right side of neck radiating to the right ear, tremor, poor health, fatigue, and tachycardia	Bilateral hypoechoic areas with ill-defined margins and hypovascularity	About 1 month	Prednisolone 30 mg/day, then Euthyrox 50 µg/day	About 2 months	Clinical relief in 10 days, neck discomfort and tenderness in the last follow-up

**(19)**	48	Guven	Sore throat, odynophagia, fever, and painful, tender, and swollen neck	Heterogeneous, patchy infiltrations and bilateral hypoechoic areas	About 20 days	Methylprednisolone 32 mg	2 months	Clinical and TFT normalization after one week
**(20)**	49	Chong et al.	Anterior neck pain and tenderness, fatigue, chills, palpitations, postural tremors, heat intolerance, anorexia, and weight loss	Diffuse hypoechoic thyroid	About 1 month	Oral aspirin and propranolol for a week	70 days	Hypothyroidism after three weeks that was managed with levothyroxine (1.6 µg/kg/day)
**(21)**	50	Semikov et al.	Weakness, myalgia, palpitations, sweating, tachycardia, and swelling and pain in the anterior surface of the neck	Diffuse thyromegaly, bilateral multiple hypoechoic areas, and right lobe pseudo-nodule	34 days	Prednisone 30 mg/day for 4 weeks with a reduction of 5 mg per week	4 weeks	Symptomatic relief within a week
**(21)**	51	Semikov et al.	Neck pain, palpitations, fever, and tachycardia	Normal	30 days	Prednisone 30 mg/day with a reduction of 5 mg per week	30 days	Symptomatic relief within a week
**(22)**	52	Kong and La Porteb	Painful and tender goiter, weight loss, malaise, and heat intolerance.	Hypoechoic and hypovascular areas	2 weeks	Propranolol 40 mg/day, paracetamol, and ibuprofen	12 weeks	Symptomatic relief and TFT normalization in six and 12 weeks, respectively.
**(23)**	53	Tjønnfjord et al.	Fever, dysphagia, cough, headache, and myalgia	Slight diffuse thyromegaly with discrete surrounding edema and heterogeneous parenchyma and hyperemia	3 weeks	Prednisolone 20 mg/day with tapering over one month	2 months	Symptomatic relief within two days
**(24)**	54	Álvarez Martín et al.	Low-grade fever, malaise, insomnia, neck pain, and grade 1 goiter	Enlarged, heterogeneous thyroid gland with normal vascularization and a left thyroid nodule	NR	NSAID then prednisone 40 mg/day with tapering over 6 weeks	3 months	TFT normalized at 3 months.
**(25)**	55	Davoodi et al.	Fever, chills, sweating, sore throat, dry cough, and slight tenderness in the thyroid area	Heterogeneous thyroid gland with bilateral ill-defined hypoechoic areas	8 days	Dexamethasone 4 mg/8 h for 5 days, then oral prednisone 25 mg/day with taper	45 days	TFT normalization at the last follow-up
**(26)**	56	Stasiak et al.	Palpable hard neck, fever, and headaches	hypoechoic areas in the right thyroid lobe	Simultaneously	Prednisone	10 months	Rapid improvement of symptoms and gradual normalization of inflammatory parameters

**(26)**	57	Stasiak et al.	NR	hypoechoic areas in the right thyroid lobe	4 weeks	Prednisone (starting dose 60 mg/day)	8 months	Recurrence of symptoms occurred after attempts of tapering of prednisolone. Prednisolone withdrawal was achieved within four months from the start of the treatment.
**(26)**	58	Stasiak et al.	Neck pain and fever	Bilateral hypoechoic areas	5 weeks	Prednisone	NR	Symptoms resolved after the completion of prednisone therapy and recurred a week after that. Thus, a re-treatment with prednisone was initiated.
**(26)**	59	Stasiak et al.	Neck pain radiating to the ear, fever, tachycardia, and mood changes	Bilateral hypoechoic areas	4 months	Prednisone 40 mg/day	NR	Complete recovery at the end of the treatment with prednisone
**(27)**	60	Brancatella et al.	Neck pain, fever, palpitations, asthenia, and anorexia	Thyromegaly, hypoechoic areas, and absent vascularization	16 days	Prednisone 25 mg/day	6 weeks	TFT normalization within six weeks
**(27)**	61	Brancatella et al.	Neck pain, fever, palpitations, asthenia, and sweating	hypoechoic areas and reduced vascularization	30 days	Prednisone 25 mg/day with taper, propranolol 40 mg/day	6 weeks	Neck pain and fever disappeared within three days and the other symptoms within two weeks. Subclinical hypothyroidism occurred after 6 weeks.
**(27)**	62	Brancatella et al.	Severe neck pain radiating to the jaw, fever, palpitations, and anxiety	Thyromegaly and hypoechoic areas	20 days	Prednisone 25 mg/day	6 weeks	Symptomatic relief within two weeks. TFT was normal at the last follow-up while taking 20 mg/d prednisone.
**(28)**	63	Sato et al.	Fever, anterior neck pain radiating to the jaw, enlargement of the left lobe of thyroid with pain spreading to the right lobe	Hypoechoic areas	23 days	Prednisolone 15 mg/day (tapered over 7 weeks)	7 weeks	The neck pain and fever resolved the next day, and the other symptoms improved within a week. TFT and Inflammatory markers normalized within seven weeks.
**(29)**	64	San Juan et al.	Neck pain radiating to submandibular region	Enlarged right lobe with ill-defined hypoechoic areas	Simultaneously	Celecoxib	8 weeks	Symptomatic relief after a month and then overt hypothyroidism after eight weeks that was managed with levothyroxine

**(30)**	65	Ruggeri et al.	Pain and tenderness in the anterior neck, fatigue, tremor, and palpitations	Thyromegaly and hypoechoic areas	6 weeks	Prednisone 25 mg/day with taper	4 weeks	Normalization of inflammatory indices and TFT within 2 and 4 weeks, respectively.
**(31)**	66	Mehmood et al.	Odynophagia, dysphagia, intermittent fever, tachycardia, dyspnea, and weight loss	Thyromegaly	7 weeks	Prednisone 20 mg/day and atenolol 25 mg/day, which were increased to 40 and 50 mg daily, respectively, after three days. After a six week taper period of prednisolone, Atenolol was also discontinued.	10 weeks	clinical recovery and TFT normalization after 10 weeks of follow up
**(32)**	67	Seyed Resuli and Bezgal	Neck pain, odynophagia, palpitations, warm sensation	Bilateral hypoechoic areas	NR	Aspirin (approximately 3 g/d) for 5 days	10 weeks	Laboratory results returned to normal 15 days after starting aspirin treatment and symptoms disappeared.
**(32)**	68	Seyed Resuli and Bezgal	Neck pain radiating to the right ear, palpitations, and sweating	Diffuse hypoechoic growth and reduced vascularization in the right lobe	NR	Aspirin (approximately 3 g/d) for 5 days	10 weeks	Laboratory results returned to normal 25 days after starting aspirin treatment and symptoms disappeared.
**(32)**	69	Seyed Resuli and Bezgal	Neck pain radiating to the left ear, weakness, irritability, and fever	Thyromegaly and hypoechoic areas	NR	Aspirin (approximately 3 g/d) for 5 days, prednisolone 1 mg/kg/day with tapering over 10 days	10 weeks	Laboratory results returned to normal on day 29 and symptoms disappeared.
**(32)**	70	Seyed Resuli and Bezgal	Neck pain radiating to both ears, irritability, and palpitations	Hypoechoic thyroid	NR	Aspirin (approximately 3 g/d) for 5 days	10 weeks	Laboratory results returned to normal 22 days after starting aspirin treatment and symptoms disappeared.
**(32)**	71	Seyed Resuli and Bezgal	Neck pain, irritability, weight loss, warm sensation, fever, and sweating	Thyromegaly and hypoechoic areas in the left lobe	NR	Aspirin (approximately 3 g/d) for 5 days, prednisolone 1 mg/kg/day with tapering over 10 days	10 weeks	Laboratory results returned to normal on day 34 and symptoms disappeared.
**(33)**	72	Bahçecioğlu et al.	Neck pain, asthenia, sweating, and palpitations	Bilateral involvement	78 days	Methylprednisolone 24 mg/day for 6 weeks	NR	NR

**(33)**	73	Bahçecioğlu et al.	Neck pain and asthenia	Unilateral involvement	21 days	Methylprednisolone 24 mg/day for five weeks, after refraction to the initial two-week treatment with NSAIDs	NR	NR
**(33)**	74	Bahçecioğlu et al.	Neck pain, asthenia, sweating, and palpitations	Bilateral involvement	49 days	Methylprednisolone 16 mg/day for four weeks, after refraction to the initial one-week treatment with NSAIDs	NR	NR
**(33)**	75	Bahçecioğlu et al.	Neck pain	Bilateral involvement	168 days	NSAIDs for 4 weeks	NR	NR
**(33)**	76	Bahçecioğlu et al.	Neck pain and sweating	Bilateral involvement	168 days	Methylprednisolone 32 mg for seven weeks, after refraction to the initial one-week treatment with NSAIDs	NR	NR
**(33)**	77	Bahçecioğlu et al.	Neck pain and weight loss	Unilateral involvement	NR	None	NR	NR
**(33)**	78	Bahçecioğlu et al.	Neck pain, asthenia, and fever	Bilateral involvement	simultaneously	Methylprednisolone 32 mg/day for 6 weeks	NR	NR
**(33)**	79	Bahçecioğlu et al.	Neck pain, sweating, cough, and palpitations	Bilateral involvement	simultaneously	Methylprednisolone 16 mg/day for 4 weeks	NR	NR
**(33)**	80	Bahçecioğlu et al.	Neck pain, fever, cough, and dyspnea	Bilateral involvement	simultaneously	Methylprednisolone 32 mg/day, given in four treatment courses due to clinical relapse; in the fourth course, colchicine 1 mg/day was added when the patient was receiving 8mg of methylprednisolone in the tapering period and was continued for 9 weeks.	NR	NR

**(34)**	81	Elawady et al.	Swollen and painful neck, fever, malaise, and myalgia	Thyromegaly and hypoechoic areas in the isthmus	2 weeks	Oral prednisone 40 mg/day and ibuprofen 800 mg/day for several weeks	2 weeks	Recovery within two weeks
**(35)**	82	Jakovac et al.	NR	NR	NR	NR	4 hours	Death due to acute respiratory distress syndrome within four hours after admission
**(36)**	83	Salehi et al.	Diffuse neck pain radiating to the jaw and hot flashes	Thyromegaly and hypoechoic areas	15 days	Aspirin, then prednisolone 25 mg/day due to unresponsiveness to aspirin	1 month	Full recovery in three weeks
**(37)**	84	Hajósi-Kalcakosz et al.	Neck pain and tenderness radiating to the jaw, fever, fatigue, myalgia, palpitation, and tremor	Diffuse hypoechoic left lobe and a small hypoechoic area in the right lobe with slurred borders	5 weeks	Methylprednisolone 32 mg/day for 2 weeks (tapered over 6 weeks), unresponsive to initial NSAID treatment	2 months	Symptomatic recovery within two weeks
**(38)**	85	Ashraf et al.	Fever, fatigue, delirium, staring, weight loss, thyroid tenderness, tachypnea, and odynophagia	NR	6 days	Intravenous dexamethasone 4 mg twice daily for 3 days, then oral prednisolone 15 mg twice daily for 1 week (tapered over 17 days) and naproxen	3 weeks	Symptomatic relief and TFT normalization within four days and three weeks, respectively.
**(39)**	86-96	Mondal et al.	Palpitations, tremor, diarrhea, fever, congestive heart failure, and neck pain and tenderness	Diffuse thyromegaly (n=7, 63.6%), hypoechoic areas (n=11, 100%) and/or reduced vascularity (n=4, 36.4%)	Mean: 23.8 days (SD: 14.8)	Prednisolone 1 mg/kg/day for 10 days for four patients, with tapering over a mean duration of 4.5 weeks; oral levothyroxine (1.6 µg/kg daily) for a patient with overt hypothyroidism after three months	6 months	Glucocorticoids did not change the outcome. At 3 months, four patients were hypothyroid (three subclinical, one overt). Two were euthyroid at 6 months, while the two other had subclinical and overt hypothyroidism. The latter two patients were both treated with oral glucocorticoids and were positive for Anti-TPO antibody.
**(40)**	97	Kleim et al.	Fever for three weeks	Right lobe enlargement with blurred borders and hypoechoic areas	NR	Prednisolone 50 mg/day for seven days with tapering over 10 weeks	5 months and 2 weeks	Immediate clinical improvement; ultrasonography and TFT normalized within five days.

**(41)**	98	Al-Shammaa and Abdlkadir	Palpitations, sweating, agitation, neck pain, tremor, and tachycardia	NR	10 days	Beta-blockers and low dose analgesia	3 months	Full recovery within 3 months
**(42)**	99	Dolkar et al.	Abdominal pain and tachycardia	NR	Simultaneously	Dexamethasone	NR	NR
**(43)**	100	Poudel Jaishi et al.	Sweating, fatigue, sore throat, dysphagia, diarrhea, insomnia, anorexia, tachycardia, neck pain, and agitation (the symptoms cannot be distinguished from COVID-19 symptoms because they coincided)	NR	Simultaneously	Steroids for 15 days	15 days	Symptomatic relief within 15 days

NR: not reported; SD: standard deviation; IQR: interquartile range; n: number; h: hour(s); TFT: thyroid function test; FT4: free T4; FT3: free T3; TSH: thyroid stimulating hormone; Anti-TPO: thyroid peroxidase antibody; NSAID: non-steroidal anti-inflammatory drug; ESR: erythrocyte sedimentation rate; IU: international unit.

Supplementary Table 4. Biochemical results of the patients

Reference	Case Number	Author	TSH (mIU/L)	Free T4	Normal Free T4 range	Free T3	Normal Free T3 range	ESR (mm/hr)	CRP (mg/L)	Tg	Anti-Tg (IU/ml)	Anti-TPO (IU/ml)	TSI	TRAb (IU/ml)
**(1)**	1	Feghali et al.	0.01	1.9 ng/dl	0.8-1.8 ng/dl	NR	NR	NR	NR	2.4 ng/mL	3	69	89%	1 IU/L
**(2)**	2	Sohrabpour et al.	0.07	19.5 pmol/L	12-21 pmol/L	18.9 pmol/L	3.1-6.8 pmol/L	25	70	NR	NR	NR	NR	NR
**(2)**	3	Sohrabpour et al.	0.01	22.3 pmol/L	12-21 pmol/L	25.4 pmol/L	3.1-6.8 pmol/L	38	56	NR	NR	NR	NR	NR
**(2)**	4	Sohrabpour et al.	0.12	24.7 pmol/L	12-21 pmol/L	19.3 pmol/L	3.1-6.8 pmol/L	18	45	NR	NR	NR	NR	NR
**(2)**	5	Sohrabpour et al.	0.01	21.9 pmol/L	12-21 pmol/L	23.7 pmol/L	3.1-6.8 pmol/L	43	83	NR	NR	NR	NR	NR
**(2)**	6	Sohrabpour et al.	0.17	26.7 pmol/L	12-21 pmol/L	21.6 pmol/L	3.1-6.8 pmol/L	51	76	NR	NR	NR	NR	NR
**(2)**	7	Sohrabpour et al.	0.23	18.4 pmol/L	12-21 pmol/L	18.1 pmol/L	3.1-6.8 pmol/L	23	39	NR	NR	NR	NR	NR
**(3)**	8	Osorio et al.	0.01	1.85 ng/dL	0.7-1.48 ng/dl	TF3 643.4 ng/dl	TF3 35-193 ng/dl	NR	NR	NR	17	15	NA	0.56
**(4)**	9	Ippolito et al.	0.08	24.6 pg/ml	0.3-17 pg/ml	5.5 pg/ml	2-4.4 pg/ml	NR	NR	187 µg/L	Negative	Negative	NR	Negative
**(5)**	10	Ünübol et al.	0.139	1.39 ng/dl	0.7-1.4 ng/dl	NR	NR	78	45	NR	Negative	Negative	NR	NR
**(5)**	11	Ünübol et al.	0.99	1.02 ng/dl	0.7-1.4 ng/dl	NR	NR	68	18.4	NR	Negative	Negative	NR	NR
**(6)**	12	Ayhan and Guner	0.01	3.72 ng/dl	0.89-1.76 ng/dl	11 ng/L	2.3-4.2 ng/L	79	74.9	NR	<1.3	NR	NR	Negative
**(7)**	13-30	Brancatella et al.	0.1	24.3 nmol/L	6-16 nmol/L	5.8 pmol/L	2.3-4.2 pmol/L	43	3.8	69 (IQR)	65 (IQR)	35 (IQR)	NR	NR
**(8)**	31	de la Higuera Lopez-Frias et al.	0.008	27.93 pmol/L	12-22 pmol/L	FT3 2.57	FT3 1.3-3.1 nmol/L	31	1.05	NR	3.4	15.5	NR	<0.10 IU/L
**(9)**	32	Khatri et al.	0.018	NR	NR	NR	NR	107	36.4	NR	NR	↑96.71	<0.1 IU/L	<1.10 IU/L
**(10)**	33	Ruano et al.	0.001	37.5 pmol/L	7-16 pmol/L	NR	NR	116	176	NR	Negative	Negative	NR	Negative

**(11)**	34	Baykan and Carlioglu	0.11	2.21	0.89-1.76 g/dL	4.19	2.3-4.2 pg/mL	52	36	NR	27	14	<0.1 IU/L	NR
**(11)**	35	Baykan and Carlioglu	0.05	2.89	0.89-1.76 g/dL	4.79	2.3-4.2 pg/mL	72	48	NR	48	2.53	<0.1 IU/L	NR
**(11)**	36	Baykan and Carlioglu	0.06	2.35	0.89-1.76 g/dL	4.28	2.3-4.2 pg/mL	42	12	NR	18	18.6	<0.1 IU/L	NR
**(11)**	37	Baykan and Carlioglu	0.21	2.13	0.89-1.76 g/dL	4.48	2.3-4.2 pg/mL	27	13.9	NR	21	7.5	<0.1 IU/L	NR
**(11)**	38	Baykan and Carlioglu	0.39	1.27	0.89-1.76 g/dL	3.45	2.3-4.2 pg/mL	50	46.7	NR	11	28	<0.1 IU/L	NR
**(12)**	39	Abreu et al.	NR	NR	NR	NR	NR	NR	NR	NR	NR	NR	NR	NR
**(12)**	40	Abreu et al.	NR	1.8 ng/dl	NR	NR	NR	NR	NR	NR	NR	NR	NR	NR
**(12)**	41	Abreu et al.	NR	NR	NR	NR	NR	NR	NR	NR	NR	NR	NR	NR
**(13)**	42	Mathews et al.	0.029	2.1 ng/dL	0.8-1.7 ng/dL	NR	NR	37	53	NR	Normal	Normal	Norm al	Normal
**(14)**	43	Brancatella et al.	0.04	27.2	11-23 nmol/L	8.7	4.6-.8.4 pmol/L	90	6.9	5.6 µg/L	120.2	<10	NR	<1.5
**(15)**	44	Chakraborty et al.	0.005	20.11 µg/dl	5.1-14.1 µg/dl	2.88 ng/ml	0.8-2 ng/ml	110	16.6	NR	NR	Negative	NR	Negative
**(16)**	45	Campos-Barrera et al.	Undetectable	1.6 ng/dl	NR	TF3<br>211 ng/dl	NR	72	66	Negative	Negative	NR	NR	NR
**(17)**	46	Mattar et al.	0.01	41.8 pmol/L	8.8-14.4 pmol/L	13.4 pmol/L	3.2-5.3 pmol/L	NR	122	NR	NR	Negative	NR	Negative
**(18)**	47	Timofeeva et al.	0.13	1.82 pg/dl	0.89-1.52 pg/dl	NR	2.43-3.98 pg/ml	24	3	NR	NR	NR	NR	NR
**(19)**	48	Guven	0.005	3.61 ng/dL	NR	4.24 pg/mL	NR	80	7.69	NR	Negative	Negative	NR	Negative
**(20)**	49	Chong et al.	0.01	2.3 ng/dl	0.6-1.3 ng/dl	FT3<br>202 ng/dl	FT3<br>80-150 ng/dl	31	14	NR	NR	1 IU/l	0.1 IU/l	NR
**(21)**	50	Semikov et al.	0.005	3.2 ng/dl	NR	NR	NR	32	NR	NR	NR	2	NR	NR
**(21)**	51	Semikov et al.	0.0083	3.4 ng/dl	NR	NR	NR	47	NR	NR	NR	1	0.73 IU/L	NR

**(22)**	52	Kong and La Porteb	0.009	26.3 pmol/L	7-16 pmol/L	7.4 pmol/L	3.8-6 pmol/L	104	171	NR	NR	Positive	NR	<0.3 IU/L
**(23)**	53	Tjønnfjord et al.	0.01	27.8 pmol/L	9-19 pmol/L	7.5 pmol/L	2.6-5.7 pmol/L	NA	86	NR	NR	<3	NR	<0.9 IU/L
**(24)**	54	Álvarez Martín et al.	0.11	2.18 ng/dl	NR	NR	NR	68	NR	NR	NR	Positive	NR	NR
**(25)**	55	Davoodi et al.	0.001	NR	NR	NR	NR	84	46	NR	NR	Negative	NR	Negative
**(26)**	56	Stasiak et al.	1.53	0.96 ng/Dl	0.93-1.7 ng/dL	3.75 pg/ml	2-4.4 pg/ml	52	9	NR	21.6	8.6	NR	1.58 IU/L
**(26)**	57	Stasiak et al.	0.005	7.7 ng/Dl	0.93-1.7 ng/dL	21.6 pg/ml	2-4.4 pg/ml	59	60	NR	13.3	10.2	NR	<0.8 IU/L
**(26)**	58	Stasiak et al.	0.01	2.39 ng/Dl	0.93-1.7 ng/dL	5.13 pg/ml	2-4.4 pg/ml	140	4.98	NR	512	10.4	NR	0.8 IU/L
**(26)**	59	Stasiak et al.	0.07	2.34 ng/dL	0.93-1.7 ng/dL	5.24 pg/ml	2-4.4 pg/ml	117	4.71	NR	25	9.4	NR	0.94 IU/L
**(27)**	60	Brancatella et al.	0.1	29.3 nmol/L	NR	8 pmol/L	NR	74	11.2	75.3 µg/l	<30	<10	NR	<1.5
**(27)**	61	Brancatella et al.	0.01	31.8 nmol/L	NR	8.9 pmol/L	NR	110	7.9	80 µg/l	38	<10	NR	<1.5
**(27)**	62	Brancatella et al.	0.01	27.8 nmol/L	NR	6.9 pmol/L	NR	NR	8	NR	NR	NR	NR	<1.5
**(28)**	63	Sato et al.	<0.001	3.2 ng/dL	0.8-1.9 ng/dL	7.3 pg/ml	2.2-4.1pg/ml	93	3.6	NR	NR	Negative	NR	NR
**(29)**	64	San Juan et al.	0.05	1.68 pg/ml	0.78-2.19 pg/ml	NR	NR	NR	Elevated	NR	Negative	Negative	NR	Negative
**(30)**	65	Ruggeri et al.	0.006	2.69 ng/dl	0.7-1.48 ng/dl	7.03 pg/ml	1.71-3.71 pg/ml	60	8.8	NR	Negative	Negative	Negati ve	NR
**(31)**	66	Mehmood et al.	0.01	4.4 ng/L	0.6-1.3 ng/L	TF3 374 ng/L	TF3 80-150 ng/L	84	44	188	NR	Negative	Negati ve	NR
**(32)**	67	Seyed Resuli and Bezgal	Low	26.8 pmol/L	12-22 pmol/L	8.4 pmol/L	3-6 pmol/L	65	High	NR	NR	Negative	Negati ve	NR
**(32)**	68	Seyed Resuli and Bezgal	Low	28.1 pmol/L	12-22 pmol/L	9.6 pmol/L	3-6 pmol/L	58	High	NR	NR	Negative	Negati ve	NR

**(32)**	69	Seyed Resuli and Bezgal	Low	43.1 pmol/L	12-22 pmol/L	14.2 pmol/L	3-6 pmol/L	70	High	NR	NR	Negative	Negati ve	NR
**(32)**	70	Seyed Resuli and Bezgal	Low	38.1 pmol/L	12-22 pmol/L	11.3 pmol/L	3-6 pmol/L	65	High	NR	NR	Negative	Negati ve	NR
**(32)**	71	Seyed Resuli and Bezgal	Low	43.5 pmol/L	12-22 pmol/L	16.2 pmol/L	3-6 pmol/L	80	High	NR	NR	Negative	Negati ve	NR
**(33)**	72-80	Bahçecioğlu et al.	0.015	27.9 pmol/L	7-15.96 pmol/L	8.1 pmol/L	3.99-6.71 pmol/L	47.5	49.2	NR	NR	NR	NR	NR
**(34)**	81	Elawady et al.	0.04	Normal level	NR	Normal level	NR	NR	NR	NR	NR	Negative	Negati ve	NR
**(35)**	82	Jakovac et al.	NR	NR	NR	NR	NR	NR	59.6	NR	NR	NR	NR	NR
**(36)**	83	Salehi et al.	0.29	15.8 ng/dl	5.1-14.1 ng/dl	190 ng/dl	40-181 ng/dl	121	92.6	NR	NR	NR	NR	NR
**(37)**	84	Hajósi-Kalcakosz et al.	0.046	1.60 ng/dl	0.70-1.48 ng/dl	4.10 pg/ml	1.71-3.71 pg/ml	34	16.9	NR	Negative	Negative	NR	NR
**(38)**	85	Ashraf et al.	NR	NR	NR	NR	NR	NR	NR	NR	NR	NR	NR	NR
**(39)**	86	Mondal et al.	0.001	1.59 ng/dl	0.61-1.72 ng/dl	4.5 pg/ml	2.2-4.2 pg/ml	67	99.9	NR	NR	Negative	NR	Negative
**(39)**	87	Mondal et al.	0.003	3.61 ng/dl	0.61-1.72 ng/dl	8.6 pg/ml	2.2-4.2 pg/ml	36	347.22	NR	NR	Negative	NR	Negative
**(39)**	88	Mondal et al.	0.001	2.99 ng/dl	0.61-1.72 ng/dl	0.73 pg/ml	2.2-4.2 pg/ml	85	219.58	NR	NR	Negative	NR	Negative
**(39)**	89	Mondal et al.	0.005	2.33 ng/dl	0.61-1.72 ng/dl	6.2 pg/ml	2.2-4.2 pg/ml	94	109.93	NR	NR	Negative	NR	Negative
**(39)**	90	Mondal et al.	0.001	2.2 ng/dl	0.61-1.72 ng/dl	6.5 pg/ml	2.2-4.2 pg/ml	51	284.95	NR	NR	Negative	NR	Negative
**(39)**	91	Mondal et al.	0.003	2.2 ng/dl	0.61-1.72 ng/dl	5.9 pg/ml	2.2-4.2 pg/ml	82	69.24	NR	NR	Negative	NR	Negative
**(39)**	92	Mondal et al.	0.001	2.45 ng/dl	0.61-1.72 ng/dl	5.1 pg/ml	2.2-4.2 pg/ml	80	74.5	NR	NR	Negative	NR	Negative
**(39)**	93	Mondal et al.	0.02	1.81 ng/dl	0.61-1.72 ng/dl	4.1 pg/ml	2.2-4.2 pg/ml	76	6.1	NR	NR	Negative	NR	Negative
**(39)**	94	Mondal et al.	0.001	2.1 ng/dl	0.61-1.72 ng/dl	4.8 pg/ml	2.2-4.2 pg/ml	73	154.49	NR	NR	Negative	NR	Negative
**(39)**	95	Mondal et al.	0.001	1.72 ng/dl	0.61-1.72 ng/dl	4.9 pg/ml	2.2-4.2 pg/ml	40	10.51	NR	NR	Negative	NR	Negative
**(39)**	96	Mondal et al.	0.03	1.87 ng/dl	0.61-1.72 ng/dl	5.5 pg/ml	2.2-4.2 pg/ml	85	99.9	NR	NR	Negative	NR	Negative
**(40)**	97	Kleim et al.	0.01	27.48 pmol/l	0.2-4.0 pmol/l	11.77 pmol/l	3.2-7.2 pmol/l	NR	193.9	NR	Normal	Normal	NR	NR
**(41)**	98	Al-Shammaa and Abdlkadir	0.021	31.6 pmol/L	10.3–22.7 pmol/L	13.8 pmol/L	2.76–11.5 pmol/L	NR	NR	NR	26.8	13.7	NR	1.24 IU/ L

**(42)**	99	Dolkar et al.	0.074	2.51 ng/dL	0.78-2.19 ng/dL	NR	NR	NR	NR	NR	<1.0	10	<0.10 IU/L	NR
**(43)**	100	Poudel Jaishi et al.	2.4 pg/ml	21 pg/ml	8-17.2 pg/ml	3.4 pg/ml	2.4-4.2 pg/ml	46	60	NR	NR	NR	NR	NR

NR: not reported; TSH: Thyroid stimulating hormone; ESR: Erythrocyte sedimentation rate; CRP: C-reactive protein; Tg: Thyroglobulin; anti-Tg: Thyroglobulin antibody; anti-TPO: Thyroid peroxidase antibody; TSI: Thyroid-stimulating immunoglobulin; TRAb: Thyrotropin receptor antibody

References:

1. Feghali K, Atallah J, Norman C. Manifestations of thyroid disease post COVID-19 illness: Report of Hashimoto thyroiditis, Graves' disease, and subacute thyroiditis. J Clin Transl Endocrinol Case Rep. 2021;22:100094.

2. Sohrabpour S, Heidari F, Karimi E, Ansari R, Tajdini A, Heidari F. Subacute Thyroiditis in COVID-19 Patients. Eur Thyroid J. 2021;9(6):321-3.

3. Osorio Martínez A, González-Razo VT, Navarro-Sánchez V, Souto Meiriño CA, Ahumada-Ayala M. SARS-CoV-2-Related Subacute Thyroiditis, Myocarditis, and Hepatitis After Full Resolution of COVID-19 Serum Markers. Am J Case Rep. 2021;22:e932321.

4. Ippolito S, Dentali F, Tanda ML. SARS-CoV-2: a potential trigger for subacute thyroiditis? Insights from a case report. J Endocrinol Invest. 2020;43(8):1171-2.

5. Ünübol M, Erdemir Z, Çolak G, Ünsal MG, Güney E. Subacute thyroiditis associated with COVID-19 infection: Two case reports. Turkish Journal of Endocrinology and Metabolism. 2021;25(3):344-8.

6. Ayhan M, Guner R. A subacute thyroiditis case after SARS-CoV-2 infection: A case report and current literature review. Haseki Tip Bulteni. 2021;59:54-6.

7. Brancatella A, Viola N, Rutigliano G, Sgrò D, Santini F, Latrofa F. Subacute Thyroiditis During the SARS-CoV-2 Pandemic. J Endocr Soc. 2021;5(10):bvab130.

8. de la Higuera López-Frías M, Perdomo CM, Galofré JC. Subacute thyroiditis following COVID-19 infection. Rev Clin Esp. 2021;221(6):370-2.

9. Khatri A, Charlap E, Kim A. Subacute Thyroiditis from COVID-19 Infection: A Case Report and Review of Literature. Eur Thyroid J. 2021;9(6):324-8.

10. Ruano R, Zorzano-Martinez M, Campos A, Rius F, Hernández M. Subacute thyroiditis might be a complication triggered by SARS-CoV-2. Endocrinol Diabetes Nutr (Engl Ed). 2020;68(10):755-6.

11. Baykan EK, Carlioglu A. Subacute Thyroiditis Secondary to SARS-CoV-2 Infection; Case Studies from Turkey. Turkish Journal of Endocrinology and Metabolism. 2021;25(2):232-7.

12. Abreu R, Miguel R, Saieg M. Subacute (De Quervain) thyroiditis during the COVID-19 pandemic. Cancer Cytopathol. 2021;129(11):844-6.

13. Mathews SE, Castellanos-Diaz J, Srihari A, Kadiyala S, Leey-Casella J, Ghayee HK, et al. Subacute Thyroiditis and Heart Failure in a Patient Presenting With COVID-19. J Investig Med High Impact Case Rep. 2021;9:23247096211009412.

14. Brancatella A, Ricci D, Viola N, Sgrò D, Santini F, Latrofa F. Subacute Thyroiditis After Sars-COV-2 Infection. J Clin Endocrinol Metab. 2020;105(7).

15. Chakraborty U, Ghosh S, Chandra A, Ray AK. Subacute thyroiditis as a presenting manifestation of COVID-19: a report of an exceedingly rare clinical entity. BMJ Case Rep. 2020;13(12).

16. Campos-Barrera E, Alvarez-Cisneros T, Davalos-Fuentes M. Subacute Thyroiditis Associated with COVID-19. Case Rep Endocrinol. 2020;2020:8891539.

17. Mattar SAM, Koh SJQ, Chandran SR, Cherng BPZ. Subacute thyroiditis associated with COVID-19. Bmj Case Reports. 2020;13(8).

18. Timofeeva LA, Aleksandrov YK, Aleshina TN, Yusova MA. Subacute thyroiditis associated with covid-19. Russ Electron J Radiol. 2021;11(3):15-24.

19. Guven M. Subacute thyroiditis in the course of coronavirus disease 2019: A case report. J Endocrinol Metab. 2020;10(3-4):110-2.

20. Chong WH, Shkolnik B, Saha B, Beegle S. Subacute Thyroiditis in the Setting of Coronavirus Disease 2019. Am J Med Sci. 2020.

21. Semikov VI, Aghayan DL, Shulutko AM, Khorobrykh TV, Aleksandrov YK, Mansurova GT, et al. Subacute thyroiditis after SARS-CoV-2 infection. Clin Case Rep. 2021.

22. Kong M, Sarah La P. Case Report: De Quervain's Thyroiditis as a Long-Term Sequelae Complication to SARS-CoV-2 Infection. Case Reports in Acute Medicine. 2021:64-70.

23. Tjønnfjord E, Moe RB, Ghanima W, Aballi S. Subacute thyroiditis after COVID-19. Tidsskr Nor Laegeforen. 2021;141(10).

24. Álvarez Martín MC, Del Peso Gilsanz C, Hernández López A. Subacute De Quervain thyroiditis after SARS-CoV-2 infection. Endocrinol Diabetes Nutr (Engl Ed). 2020;68(10):754.

25. Davoodi L, Oladi Z, Jafarpour H, Zakariaei Z, Soleymani E, Razavi A. A 33-year-old man with COVID-19 presented with subacute thyroiditis: A rare case report and literature review. New Microbes New Infect. 2021;41:100871.

26. Stasiak M, Zawadzka-Starczewska K, Lewiński A. Clinical Manifestation of Subacute Thyroiditis Triggered by SARS-CoV-2 Infection Can Be HLA-Dependent. Viruses. 2021;13(12).

27. Brancatella A, Ricci D, Cappellani D, Viola N, Sgrò D, Santini F, et al. Is Subacute Thyroiditis an Underestimated Manifestation of SARS-CoV-2 Infection? Insights From a Case Series. J Clin Endocrinol Metab. 2020;105(10).

28. Sato D, Nishiguchi S, Tanaka E. Successful Management of Subacute Thyroiditis Following SARS-CoV-2 Infection. Intern Med. 2021;60(22):3573-6.

29. San Juan MDJ, Florencio MQV, Joven MH. SUBACUTE THYROIDITIS IN A PATIENT WITH CORONAVIRUS DISEASE 2019. AACE Clin Case Rep. 2020.

30. Ruggeri RM, Campennì A, Siracusa M, Frazzetto G, Gullo D. Subacute thyroiditis in a patient infected with SARS-COV-2: an endocrine complication linked to the COVID-19 pandemic. Hormones (Athens). 2021;20(1):219-21.

31. Mehmood MA, Bapna M, Arshad M. A Case of Post-COVID-19 Subacute Thyroiditis. Cureus. 2020;12(12):e12301.

32. Seyed Resuli A, Bezgal M. Subacute Thyroiditis in COVID-19 Patients. Ear Nose Throat J. 2021:1455613211012114.

33. Bahçecioğlu AB, Karahan ZC, Aydoğan BI, Kalkan IA, Azap A, Erdoğan MF. Subacute thyroiditis during the COVID-19 pandemic: a prospective study. J Endocrinol Invest. 2022;45(4):865-74.

34. Elawady SS, Phuyal D, Shah RK, Mirza L. A Case of Subacute Thyroiditis following COVID-19 Infection. Case Rep Endocrinol. 2022;2022:2211061.

35. Jakovac H, Ferencic A, Stemberger C, Mohar Vitezic B, Cuculic D. Detection of Sars-Cov-2 antigens in thyroid gland showing histopathological features of subacute thyroiditis. Eur Thyroid J. 2022.

36. Salehi AM, Salehi H, Mohammadi HA, Afsar J. SARS-CoV-2 and Subacute Thyroiditis: A Case Report and Literature Review. Case Rep Med. 2022;2022:6013523.

37. Hajósi-Kalcakosz S, Dénes J, Góth M. Subacute thyroiditis associated with COVID-19 infection: a report of an increasing entity. Arch Endocrinol Metab. 2022;66(1):118-28.

38. Ashraf S, Imran MA, Ashraf S, Hafsa H-T, Khalid S, Akram MK, et al. COVID-19: A Potential Trigger for Thyroid Dysfunction. The American journal of the medical sciences. 2021;362(3):303-7.

39. Mondal S, Dasgupta R, Lodh M, Ganguly A. Subacute thyroiditis following recovery from COVID-19 infection: Novel clinical findings from an Eastern Indian cohort. Postgraduate Medical Journal. 2022.

40. Kliem T, Strobel D, Heinke F, Pavel M, Neurath MF, Neufert C. Case report: persistent fever after SARS-CoV-2 infection. Internist. 2022.

41. Al-Shammaa MS, Abdlkadir AS. A case of post COVID-19 subacute thyroiditis. Clin Case Rep. 2022;10(7):e6092.

42. Dolkar T, Jitidhar F, Patel MJ, Hamad AM, Salauddin F, Shiferaw-Deribe Z, et al. Painless Subacute Thyroiditis in a Patient With Acute COVID-19 Infection: A Transient Event. Cureus. 2022;14(7):e26924.

43. Poudel Jaishi P, Neupane SK, Neupane PK, Koirala D, Kandel A, Joshi K. Case report: Paradoxical manifestation of covid 19 induced thyroiditis in the unvaccinated patient. Ann Med Surg (Lond). 2022;80:104204.
